# Fertility Preservation Discussions Between Young Adult Rectal Cancer Survivors and Their Providers: Sex-Specific Prevalence and Correlates

**DOI:** 10.1093/oncolo/oyac052

**Published:** 2022-04-15

**Authors:** Julia Stal, Serena Y Yi, Sally Cohen-Cutler, Phuong Gallagher, Afsaneh Barzi, David R Freyer, Joel E Milam, Heinz-Josef Lenz, Kimberly A Miller

**Affiliations:** Department of Population and Public Health Sciences, Keck School of Medicine of USC, Los Angeles, CA, USA; Department of Population and Public Health Sciences, Keck School of Medicine of USC, Los Angeles, CA, USA; Cancer and Blood Disease Institute, Children’s Hospital Los Angeles, Los Angeles, CA, USA; The Colon Club, USA; Department of Medical Oncology and Therapeutics Research, City of Hope National Medical Center, Duarte, CA, USA; Department of Population and Public Health Sciences, Keck School of Medicine of USC, Los Angeles, CA, USA; Cancer and Blood Disease Institute, Children’s Hospital Los Angeles, Los Angeles, CA, USA; Department of Pediatrics, Keck School of Medicine of the University of Southern California, Los Angeles, CA, USA; University of Southern California Norris Comprehensive Cancer Center, Los Angeles, CA, USA; School of Medicine, Department of Epidemiology and Biostatistics, Chao Family Comprehensive Cancer Center, University of California, Irvine, CA, USA; Division of Oncology, University of Southern California Norris Comprehensive Cancer Center, Los Angeles, CA, USA; Department of Population and Public Health Sciences, Keck School of Medicine of USC, Los Angeles, CA, USA; Department of Dermatology, Keck School of Medicine of the University of Southern California, Los Angeles, CA, USA

**Keywords:** cancer, rectal cancer, fertility, reproductive health, survivorship

## Abstract

**Background:**

Young adults (YA) diagnosed with rectal cancer are disproportionately impacted by the gonadotoxic effects of treatment and potential subsequent infertility.

**Objective:**

The purpose of this study was to characterize the prevalence of fertility preservation measures used, reasons why such measures were not used, and correlates of discussion between providers and YA rectal cancer survivors.

**Design:**

An online, cross-sectional survey was administered on the Facebook page of a national colorectal cancer (CRC) advocacy organization. Eligible participants were rectal cancer survivors diagnosed before age 50, between 6 and 36 months from diagnosis or relapse, and based in the US.

**Results:**

Participants were 148 rectal cancer survivors. Over half of the survivors reported that their doctor did not talk to them about potential therapy-related fertility complications. Only one-fifth of survivors banked sperm (males) or eggs/embryos (females) prior to their cancer therapy. Older age at diagnosis and greater quality of life were significantly associated with a higher likelihood of fertility discussions among males. Greater quality of life was significantly associated with a higher likelihood of fertility discussion among females.

**Conclusions:**

These findings indicate that the majority of YA rectal cancer survivors do not receive, or cannot recall, comprehensive cancer care, and help to identify patients with rectal cancer who may be at risk for inadequate fertility counseling. Clinicians should provide proper counseling to mitigate this late effect and to ensure optimal quality of life for YA rectal cancer survivors.

## Teaser

Young adults diagnosed with rectal cancer are disproportionately affected by the gonadotoxic effects of treatment and potential subsequent infertility. This article reports on the prevalence of fertility preservation measures used, reasons why such measures might not be used, and discussions between healthcare providers and survivors of rectal cancer.

**Implications for Practice**

Young adults diagnosed with rectal cancer are disproportionately affected by the gonadotoxic effects of treatment causing potential subsequent infertility. This article reports on the prevalence of fertility preservation measures used, reasons why such measures might not be used, and discussions between healthcare providers and young adult rectal cancer survivors.

## Background

The American Cancer Society (ACS) estimates 45 230 new cases of rectal cancer in the US in 2021.^1^ While colon and rectal cancer are often grouped together due to tumor location, the incidence of rectal cancer is rising faster than colon cancer and is increasing among young adults (YA).^2^ Recent findings from the US National Cancer Database indicate patients under age 50 are more likely to have a primary rectal tumor than patients over age 50.^3^ Due to advances in diagnosis and treatment, approximately 67% of patients diagnosed with rectal cancer survive at least 5 years from diagnosis, many of whom achieve normal life spans.^1,4^ Unfortunately, many long-term survivors of rectal cancer experience chronic late effects resulting from cancer treatment, including neuropathy, stool leakage, ostomy bags, urinary incontinence, fatigue, sexual difficulties, and other psychological impacts.^5,6^ In particular, many cancer treatment modalities such as chemotherapy and radiation therapy, while effective, are gonadotoxic and have a high likelihood of subsequent infertility.^7-9^ The American Society of Clinical Oncology (ASCO) indicates that oncologists should discuss possible treatment-related infertility with all patients with cancer who are of reproductive age, defined by the World Health Organization (WHO) as age 15-49.^10,11^ Fertility discussions should include infertility risk, preservation options, referral to a reproductive specialist, costs, and pregnancy risks as deemed necessary for the patient.^10^ Such discussions are particularly relevant for YAs with cancer, who are in the prime of their reproductive years and have identified fertility-related distress following their cancer treatment as an unmet need.^12^

Because of the natural age limits of reproductive potential, the age of YAs emphasizes the need for comprehensive fertility discussions between patients and their providers.^13^ While many YA patients report a strong desire to have children in the future,^14^ only a small minority access fertility treatment.^15^ Prompt, time-sensitive fertility discussions prior to cancer therapy are required to ensure a variety of preservation options remain viable for the patient.^10^ However, studies indicate that adequate education in fertility options remains elusive in this population.^16^ For example, in one study, 44% of YA survivors were unsure if their treatment had impacted their fertility, with males being more likely to report uncertainty than females (50% males versus 39% females).^12^

Barriers to uptake of fertility preservation among YAs exist at both the provider and patient level. By training, oncologists primarily focus on cancer diagnosis and treatment, often emphasizing the urgency of treatment, affording limited time for fertility discussion and preservation.^15^ Similarly, YA survivors may choose not to preserve fertility in favor of prioritizing their cancer treatment.^17^ Thus, both oncologists and patients may view fertility preservation as secondary to treatment, despite recommended guidelines.^10^ Further barriers include financial considerations at the start of treatment, lack of insurance coverage, lack of specialist accessibility, and/or not being referred, among many others.^18^

Patients with rectal cancer often receive intensive and aggressive therapy, resulting in impairments in urinary and sexual outcomes, and are particularly vulnerable to developing treatment-related infertility.^8,19-21^ While a lack of fertility knowledge is well-described among cancer survivors,^16^ the rectal cancer population has been understudied with respect to oncofertility. The present study sought to examine the prevalence of fertility discussion and preservation, characterize reasons why fertility was not preserved, and identify correlates of discussion between providers and YA rectal cancer survivors. Consistent with prior research among YAs across cancer types, we hypothesized that there would be low rates of both fertility discussion^16^ and fertility preservation^22^ among rectal cancer survivors. Additionally, we hypothesized that greater treatment intensity, higher socioeconomic status, level of education, income, and partnered marital status would be associated with fertility discussion and fertility preservation, in alignment with prior research among other cancer types.

## Methods

An online, cross-sectional survey was administered on the Facebook page of a national colorectal cancer (CRC) advocacy organization, the Colon Club, between August 31 and September 3, 2020.^23,24^ The Colon Club Facebook page has approximately 7000 members and strives to raise awareness, educate, and help those with CRC, particularly those diagnosed as YAs and are based in the US. Eligible participants were either colon or rectal cancer survivors under age 50 at diagnosis, between 6 and 36 months from diagnosis or relapse, and based in the US. Eligibility criteria included patients within 6-36 months from diagnosis or relapse to capture patients in the early stages of cancer survivorship who have recent interactions with the healthcare system to homogenize the sample. Due to incidence rates^2^ and subsequent risk of infertility, only those reporting rectal cancer were included in the present analyses. Upon survey completion, participants received a $20 electronic gift card. The study was approved by the University of Southern California Institutional Review Board.

Using an online Research Electronic Data Capture (REDCap) survey, respondents were asked a series of questions to determine their eligibility for participation in the study. If respondents satisfied eligibility criteria, they were provided with an Information Sheet regarding the study and were asked to respond “yes” for consent to participate. Numerous steps of screening and consent were used to ensure data validity and integrity, and to reduce potential fraudulent responses encountered in social media recruitment (ie, automated software or “bots”).^25^ Steps to reduce fraudulent responses included the prohibition of duplicate email addresses; removal of respondents whose survey completion time was well below the average (defined as less than 5 min given an average completion time of 17 min); and removal of respondents reporting improbable medical treatment patterns (defined as participants diagnosed with stage 1-3 rectal cancer who did not report relapsed disease but reported receipt of immunotherapy) as determined by a medical oncologist (A.B.). Additional steps to ensure data integrity are detailed elsewhere.^23,24^

### Measures

Respondents were asked to complete a survey including a wide variety of measures encompassing clinical, psychosocial, and general demographic aspects related to fertility. The survey was only available in English as the Colon Club’s Facebook page is English-only. Gender options included *woman, man, transgender*, and *a gender not listed/other*. Respondents who identified as transgender or other were able to choose a male- or female-specific survey. In the present study, we report this as male or female, because of the sex-specific implications of fertility preservation.

### Fertility Preservation Discussion Between Patient And Provider

Both male and female survivors were asked to indicate, *“Has a doctor ever talked to you about problems with your ability to have children after your treatment?”* (Yes, no, not sure). Survivors who indicated female sex were asked, “*Did you bank eggs/embryos prior to your cancer therapy?” (Yes, no, not sure*). Survivors who indicated male sex were asked, *“Did you bank sperm prior to your cancer therapy?” (Yes, no, not sure)*. Both male and female respondents who reported “no” to the prior item were asked, *“(If “no”) I decided not to because”* with response options including, *“not sure”, “I chose not to”, “I did not know this was an option”, “I wanted to, but could not afford it*”; and “*I wanted to, but my treatment would not allow it”*. These items have been previously used in large, ethnically diverse population-based cohorts and were adapted for use by the research team.^26^

### Correlates

#### Quality of Life

The 36-item Functional Assessment of Cancer Therapy-Colorectal (FACT-C) measure was used to assess the quality of life among participants.^27^ Response options utilize a 5-point Likert-type scale and range from “*Not at all*” to “*Very much*,” with higher scores indicating the greater quality of life. Subscale domains include physical (7 items; score range 0-28), social/familial (7 items, score range 0-28), emotional (6 items; score range 0-24), and functional well-being (7 items, score range 0-28), as well as a CRC-specific subscale (9 items, score range 0-28).

#### Financial Toxicity

The 12-item Comprehensive Score for Financial Toxicity-Functional Assessment of Chronic Illness Therapy (COST-FACIT) measure was used to assess financial distress in participants.^28^ Response options utilize a 5-point Likert-type scale and range from “*Not at all*” to “*Very much*,” with total scores ranging from 0 to 44, in which higher scores indicate better financial well-being.

#### Healthcare Ratings

Survey items from the Surveillance, Epidemiology, and End Results Program-Consumer Assessment of Healthcare Providers and Systems (SEER-CAHPS) were used to examine survivor ratings of their primary healthcare provider, specialist, and health plan on a Likert-type scale ranging from 0 to 10.^29^ To examine perceptions of primary healthcare providers, survivors were asked to indicate *“How would you rate your primary healthcare provider (different from your oncologist)?”*; to examine perceptions of specialists, survivors were asked to indicate *“How would you rate your specialists?”*; and to examine health plans, survivors were asked *“How would you rate your health plan?”*.

### Statistical Analysis

Given the distinct nature of fertility preservation by sex, 2 independent models were examined, stratified by sex. The outcome variable consisted of a report of any fertility discussion (*yes* versus *no* or *not sure*). Variables considered for inclusion in the models included race/ethnicity (non-Hispanic/Latino White versus respondents of color [Hispanic/Latino/Latinx, Black or African American, Asian, Native Hawaiian or Pacific Islander]), treatment intensity (scored 0-4 based on a number of treatment modalities received [chemotherapy, immunotherapy/targeted therapies, surgery, and/or radiation]), employment (working full-time versus working part-time or less [part-time, stay at home parent, student, unemployed or disabled, or other], and education (high school graduate or less versus some college or more [eg, post-graduate training]).

Bivariate analyses were conducted with variables selected for their hypothesized significance to the outcome and to describe the sample. Variables significant at *P* < .10 were included in the multivariable logistic regression models. Stage of diagnosis and treatment intensity were included in the model due to the individual impact each of these factors may have on fertility discussions.^9^ All tests were 2-tailed, with an α criterion of *P* < .05. Statistical analysis was performed using Stata (Version 14.2, StataCorp, College Station, TX).

## Results

After eliminating respondents who did not meet eligibility criteria or who were deemed potentially fraudulent, a total of 148 rectal cancer survivors were included in the study. Table 1 provides frequencies to characterize the sample. Respondents had a mean current age of 35.1 years (±6.8; range 20-49) and a mean age of diagnosis of 33.2 years (±7.0; range 17-48). Rectal (*N* = 148) cancer survivors were diagnosed primarily with stage 2 cancer (61.6%) and were primarily treated with surgery (54.7%).

**Table 1.** Sample characteristics^a^

	Male	Female	Total
	*N* (%) or M (SD)	*N* (%) or M (SD)	*N* (%) or M (SD)
Sociodemographic factors			
Sex	96 (65.31)	51 (34.69)	148
Current age	35.56 (7.05)	34.31 (6.20)	35.05 (6.81)
Age of diagnosis	33.71 (7.25)	32.60 (6.32)	33.26 (6.97)
Race/ethnicity			
Hispanic/Latino/Latinx	11 (11.46)	4 (8.16)	15 (10.34)
White	76 (79.17)	38 (77.55)	114 (78.62)
Black or African American	7 (7.29)	6 (12.24)	13 (8.97)
Asian	1 (1.04)	.	1 (0.69)
Native Hawaiian or Pacific Islander	1 (1.04)	1 (2.04)	2 (1.38)
Marital status			
Single (never married)	19 (19.79)	12 (23.53)	32 (21.62)
Living with a partner	20 (20.83)	9 (17.65)	29 (19.59)
Married	56 (58.33)	28 (54.90)	84 (56.76)
Widowed	1 (1.04)		1 (0.68)
Divorced/separated		2 (3.92)	2 (1.35)
Employment			
Working full-time	55 (57.29)	19 (37.25)	74 (50.00)
Working part-time	34 (35.42)	27 (52.94)	62 (41.89)
Stay-at-home parent		1 (1.96)	1 (0.68)
Unemployed or permanently disabled	6 (6.25)	3 (5.88)	9 (6.08)
Other	1 (1.04)	1 (1.96)	2 (1.35)
Highest level of education			
Some high school or less (<12 years)	6 (6.25)	2 (3.92)	8 (5.41)
High school graduate or GED (12 years)	5 (5.21)	6 (11.76)	12 (8.11)
Some college training or associates degree	68 (70.83)	41 (80.39)	109 (73.65)
College graduate or more^b^	17 (17.71)	2 (3.92)	19 (12.84)
Financial toxicity score	20.79 (4.73)	19.57 (5.51)	20.35 (5.02)
Have children	48 (51.06)	23 (45.10)	71 (48.63)
Clinical factors			
*Treatment* ^c^			
Chemotherapy	32 (33.33)	16 (31.37)	48 (32.43)
Radiation	45 (46.88)	30 (58.82)	75 (50.68)
Surgery	52 (54.17)	28 (54.90)	81 (54.73)
Immunotherapy	27 (28.13)	8 (15.69)	35 (23.65)
Treatment intensity	1.63 (0.85)	1.61 (0.81)	1.62 (0.83)
Relapse	54 (56.25)	28 (54.90)	82 (55.41)
Stage of diagnosis			
Stage 1	14 (14.74)	9 (18.00)	23 (15.75)
Stage 2	58 (61.05)	32 (64.00)	90 (61.64)
Stage 3	22 (23.16)	8 (16.00)	31 (21.23)
Stage 4	1 (1.05)	1 (2.00)	2 (1.37)
Healthcare ratings			
Rating of PCP	5.96 (1.95)	5.35 (1.87)	5.77 (1.95)
Rating of specialist	6.27 (2.12)	5.82 (1.97)	6.12 (2.07)
Rating of health plan	6.25 (1.97)	5.22 (2.12)	5.90 (2.07)

Total values may not sum to *N* = 148 due to item missingness.

Includes BA/BS, MA/MS, PhD, MD, or other graduate degree.

Some respondents endorsed more than one treatment type.

Table 2 characterizes fertility discussion and preservation by respondent sex. Over half of both male and female survivors reported that their doctor did not talk to them about problems with their ability to have children after treatment. Roughly 75% of male and female survivors did not bank sperm (males) or eggs/embryos (females) prior to their cancer therapy. Of those, 15.8% of males and 23.7% of females endorsed the desire to have preserved but cited financial concerns as a deterrent. Furthermore, 21.1% of males and 15.8% of females endorsed not knowing preserving fertility was an option.

**Table 2.** Prevalence of fertility discussion and preservation

	Gender	
	Male (*N* = 96)^a^	Female (*N* = 51)^a^
Has a doctor ever talked to you about problems with your ability to have children after your treatment?		
Yes	39 (40.63)	21 (42.00)
No	54 (56.25)	28 (56.00)
Not sure	3 (3.13)	1 (2.00)
Did you bank eggs/embryos (female; sperm, male) prior to your cancer therapy?		
Yes	19 (20.00)	11 (22.45)
No	73 (76.84)	37 (75.51)
Not sure	3 (3.16)	1 (2.04)
If no (did not bank eggs/embryos or sperm), I decided not to because…		
I wanted to, but my treatment would not allow it	4 (5.26)	2 (5.26)
I wanted to, but could not afford it	12 (15.79)	9 (23.68)
I did not know this was an option	16 (21.05)	6 (15.79)
I chose not to	39 (51.32)	19 (50.00)
Not sure	5 (6.58)	2 (5.26)

Total values may not sum to *N* = 96 (males) and *N* = 51 (females) due to item missingness.

Table 3 provides bivariate and multivariable models of correlates of fertility discussions by sex. In bivariate analyses among males, older age at diagnosis, being partnered (versus no partner), higher financial toxicity score, and higher quality of life score were significantly associated with having a fertility discussion. The lower level of education (high school graduate or less versus some college or more) was significantly negatively associated with having a fertility discussion. In multivariable analysis among males, older age at diagnosis and higher quality of life score retained their significance in the adjusted model.

**Table 3.** Bivariate and multivariable models of correlates of fertility discussions among male and female rectal cancer survivors

	Male		Female	
	OR (95% CI)	AOR (95% CI)	OR (95% CI)	AOR (95% CI)
Sociodemographic factors				
2003Age of diagnosis	1.09 (1.03, 1.16)**	1.12 (1.03, 1.21)**	1.10 (0.99, 1.21)+	1.06 (0.94, 1.19)
Race/ethnicity				
White	1.03 (0.38, 2.82)	.	0.31 (0.08, 1.26)	.
Respondent of color^a^	1.0	.	1.0	.
Marital status				
Partner	3.41 (1.04, 11.17)*	3.08 (0.68, 14.05)	0.64 (0.18, 2.21)	.
No partner	1.0	1.0	1.0	.
Employment				
Working full-time	1.12 (0.49, 2.56)	.	1.43 (0.45, 4.52)	.
Working part-time or less	1.0	.	1.0	.
Level of education				
High school graduate or less	0.12 (0.02, 1.01)+	0.51 (0.05, 5.61)	0.51 (0.09, 2.90)	.
Some college or more	1.0	1.0	1.0	.
Income	1.28 (0.94, 1.73)	.	0.70 (0.44, 1.11)	.
Financial toxicity	1.09 (0.99, 1.20)+	1.06 (0.92, 1.22)	1.02 (0.92, 1.13)	.
Children				
Have children	1.90 (0.82, 4.39)	.	1.12 (0.36, 3.45)	.
Do not have children	1.0	.	1.0	.
Clinical factors				
Quality of life	1.06 (1.02, 1.11)**	1.10 (1.03, 1.17)**	1.09 (1.02, 1.16)*	1.09 (1.01, 1.17)*
Treatment intensity	0.87 (0.53, 1.41)	1.27 (0.67, 2.42)	0.58 (0.27, 1.23)	0.59 (0.24, 1.44)
Stage of diagnosis (1-4)	1.36 (0.71, 2.60)	1.70 (0.68, 4.23)	2.51 (0.91, 6.91)+	2.37 (0.72, 7.77)
Healthcare ratings				
Rating of PCP	1.08 (0.87, 1.33)	.	1.18 (0.87, 1.62)	.
Rating of specialist	1.03 (0.84, 1.25)	.	1.09 (0.81, 1.46)	.
Rating of health plan	1.10 (0.89, 1.36)	.	1.13 (0.86, 1.48)	.

Includes Hispanic/ Latino/ Latinx, Black or African American, Asian, Native Hawaiian or Pacific Islander, American Indian or Alaska Native.

+*P* < .10; *P < .05, **P <.01, ***P <.001.

In bivariate analyses among females, older age at diagnosis, higher quality of life score, and advanced stage of diagnosis (versus lower stage of diagnosis) were significantly associated with having a fertility discussion. In multivariable analysis among females, those with a higher quality of life score were significantly more likely to report a fertility discussion.

## Discussion

In the present study, we sought to characterize the prevalence of fertility discussion and preservation, identify reasons why fertility was not preserved, and identify correlates of discussion between providers and YA rectal cancer survivors, an under-researched young adult cancer population where rates of disease are sharply increasing.^30^ Overall, fertility counseling was infrequently provided to patients, reflecting a continued unmet need for YA rectal cancer survivors. The low level of fertility discussion suggests inadequate counseling for rectal cancer survivors, despite a primary tumor in the abdominopelvic region and the frequent use of gonadotoxic therapies.

According to recent SEER findings, the mean age of CRC diagnosis among adolescent and young adults (AYA) is 33.6 years (SD ± 4.8), similar to the mean age of diagnosis of the present sample (32.3 years [SD ± 7.0]).^31^ The present sample had a greater number of survivors diagnosed with the regional disease (specifically stage 2, 61.6%), and a lower number diagnosed with distant (stage 4) disease (1.4%), compared to 2009-2018 SEER findings (35% regional disease and 24% distant disease [4.6% unstaged]).^31^ In addition, the present sample contained more males than incident cases in 2010-2015 SEER findings (65.3% versus 52%, respectively).^32^ Therefore, in comparison to prior SEER findings, the stage distribution of the present sample is indicative of potential survival bias. As such, recent SEER findings have shown increases in stage 2 disease among YAs which may account for the stage distribution in the present sample.^32^

The low prevalence of fertility discussions was consistent across sex, with over half of both male and female rectal cancer survivors reporting not having a fertility discussion with their provider. Further, among rectal cancer survivors who did not preserve their fertility, approximately one-fifth were not aware of preservation options. This suggests that young rectal cancer survivors are not receiving, or cannot recall, guideline-concordant cancer care with regards to fertility discussion.^16^ Risk rates for delaying treatment to allow for fertility discussion and preservation vary. In some studies delaying treatment up to 1 year has not been shown to increase the odds of CRC-specific death,^33^ while in others, treatment is recommended to be initiated within 30 days.^34^ While further research is needed to determine the safety of delaying treatment on a risk-stratified basis, rates of fertility counseling are likely to be dependent upon tumor progression and patient need for rapid treatment. Nevertheless, fertility counseling is needed to initiate action and/or manage expectations of fertility preservation among patients. As such, future research is needed to determine variability in fertility-related counseling among low- versus high-stage patients.

Reasons for the gaps in fertility discussion and preservation are multifold. In some cases, healthcare providers may not have an adequate understanding of the fertility implications of therapy. For example, oncologists and hematologists who report low confidence in treatment-related infertility risks among male patients were less likely to counsel these patients on fertility issues.^35^ Without comprehensive knowledge of the late effects posed by cancer treatment, providers, such as oncologists, are unable to prevent and address subsequent patient outcomes. As the primary source for patient education, a lack of knowledge in gonadotoxicity and the need for fertility preservation on the part of the oncologic care team will invariably result in equally deficient patient awareness. It is essential that providers ensure patients receive timely fertility discussions to mitigate the late effects of their cancer treatment and offer patients quality, comprehensive, cancer care.

High costs result in the underutilization of fertility preservation. In our sample, approximately one-fifth of survivors endorsed a desire to preserve fertility but were unable to afford it. For females, preservation is typically a time-consuming and invasive procedure ranging from $10 000 to $15 000 with an additional $300-$600 annual cost for storage (eg, egg, embryo, or ovarian tissue freezing, ovarian transposition, or ovarian suppression).^36^ For males, preservation is typically a rapid process ranging from $500 to $12 000 with an additional $150-$500 cost for annual storage (eg, sperm banking, testicular sperm extraction, or electroejaculation).^36^ In 2019, the median income of men and women in the US who worked full-time, year-round, was approximately $57 000 and $47 000, respectively.^37^ This substantial financial burden remains a prominent barrier for the uptake of preservation.

Among males, better financial well-being was independently associated with a greater likelihood of fertility discussion. This finding suggests that providers may discuss fertility preservation options more frequently with patients who are perceived as able to afford them. High cancer treatment costs create an initial burden on the patient and fertility preservation may be seen as an impractical additional financial burden. However, despite high costs, all patients should receive fertility preservation counseling and physicians should not make medical decisions based on the suspicion that a patient cannot afford preservation. In addition to the cost, the decision-making process and procedures must frequently be completed within a relatively short time frame,^15^ eliminating the potential to save up or distribute the cost over several months, further increasing financial strain on patients and decreasing the likelihood that patients are able to afford fertility preservation. Furthermore, young cancer survivors experience more financial hardship and are more often uninsured or underinsured than older cancer survivors, making the high cost of fertility preservation even more prohibitive.^38^ For example, YAs are more likely to have competing for financial concerns such as student debt,^39^ and/or may take an unpaid leave or switch to part-time work during cancer,^40^ contributing to this multifactorial burden. Knowing this, physicians may defer discussions of fertility preservation in an effort to minimize the additive financial burden on the patient.

Both male and female respondents who were diagnosed at the older range of the age spectrum were more likely to report a fertility discussion. Those diagnosed at a younger age may be less likely to have a long-term partner or an understanding of their future goals than those diagnosed at an older age, and as such, physicians may consider fertility preservation discussions to be less relevant. In addition, older patients of reproductive age may be more concerned with fertility by virtue of the natural age limits associated with reproductive potential. As such, older patients may be more “activated” (have knowledge, skill, and confidence to manage their health^41^) and thus may be more likely to inquire about fertility preservation than younger patients. Past research indicates that patient activation and health outcomes are directionally related, such that as activation increases, health outcomes are likely to improve.^41^ While the present study did not measure patient activation, a relationship between patient age and fertility discussion was found, and further research exploring age and patient activation is needed to characterize differences among fertility discussions between older and younger patients of reproductive age.

Among both sexes, those with a higher quality of life scores were significantly more likely to report having a fertility discussion after adjusting for stage and treatment intensity. It is possible that patients with a greater quality of life are more educated about their treatment and thus are more prepared for survivorship. Those with a greater quality of life may be less likely to have impaired memory due to stress and repression and thus have greater information recall.^42^ It is also possible that fertility discussions served as a proxy for the receipt of patient-centered care, promoting well-being in survivorship and increasing patient satisfaction,^43^ while controlling for ratings of primary healthcare providers, specialists, and health plans. This area warrants further research to characterize the relationship between quality of life and fertility discussion among rectal cancer survivors.

The present study has limitations. The cross-sectional design limits the ability to draw causal inferences. While rigorous attempts to reduce fraudulent responses were made, the nature of social media sampling prevents complete verification of respondents’ patient status. Similarly, a social media sample may not be fully representative of the overall patient population as the respondents were connected to an online resource and may be more motivated to seek cancer information. Respondents of color were grouped due to approximately 78.6% of the sample being non-Hispanic/Latino White and because rectal cancer respondents of color report greater unmet needs than non-Hispanic/Latino whites,^44^ limiting power for robust subgroup analyses. We did not ask if respondent families were complete, however, we did ask if they did not want to preserve fertility. Further, we are unable to determine each respondent’s individual risk of infertility, however, each has received at least one of 4 treatment types that have been shown to affect reproductive potential (chemotherapy, immunotherapy/targeted therapies, surgery, and/or radiation).^7-9^ Additionally, we did not measure whether the patient or provider-initiated fertility counseling in the event that counseling did occur and thus could not account for respondents with greater patient activation who may be more likely to self-refer.^41^ Despite these limitations, this study fills a critical gap in the literature, as little research currently exists on rectal cancer-specific fertility preservation discussions.

Future research should ensure that survivors of color are represented in the data and should examine differences across various racial/ethnic groups. Psychosocial aspects involving the holistic burden of cancer on family planning and fertility preservation should be examined to understand gaps in patient care. In addition, among patients who report having a fertility discussion, researchers should examine the content of these discussions to determine if they are concordant with the full scope of guidelines and recommendations.^10^

## Conclusion

In summary, the findings of this study suggest that YA patients with rectal cancer are at risk for inadequate fertility counseling, and therefore, inadequate care, despite their receipt of gonadotoxic treatment(s). Providers must ensure that YA patients with rectal cancer with reproductive potential receive timely, guideline-concordant,^10^ fertility discussions to mitigate this late effect and to ensure optimal quality of life for YA rectal cancer survivors.

## Data Availability

The data underlying this article will be shared on reasonable request to the corresponding author.
